# Investigating sentence processing and working memory in patients with mild Alzheimer and elderly people

**DOI:** 10.1371/journal.pone.0266552

**Published:** 2022-11-01

**Authors:** Maryam Nasiri, Saeideh Moayedfar, Mehdi Purmohammad, Leila Ghasisin

**Affiliations:** 1 Student Research Committee, School of Rehabilitation, Isfahan University of Medical Sciences, Isfahan, Iran; 2 Department of speech therapy, School of Rehabilitation Sciences, Tehran University of Medical Sciences, Tehran, Iran; 3 Institute for Cognitive and Brain Sciences, Shahid Beheshti University, Tehran, Iran; 4 Communication Disorders Research Center, Isfahan University of Medical Sciences, Isfahan, Iran; Nathan S Kline Institute, UNITED STATES

## Abstract

**Introduction:**

Linguistic disorders are one of the common problems in Alzheimer’s disease, which in recent years has been considered as one of the key parameters in the diagnosis of Alzheimer (AD). Given that changes in sentence processing and working memory and the relationship between these two activities may be a diagnostic parameter in the early and preclinical stages of AD, the present study examines the comprehension and production of sentences and working memory in AD patients and healthy aged people.

**Methods:**

Twenty-five people with mild Alzheimer’s and 25 healthy elderly people participated in the study. In this study, we used the digit span to evaluate working memory. Syntactic priming and sentence completion tasks in canonical and non-canonical conditions were used for evaluating sentence production. We administered sentence picture matching and cross-modal naming tasks to assess sentence comprehension.

**Results:**

The results of the present study revealed that healthy elderly people and patients with mild Alzheimer’s disease have a significant difference in comprehension of relative clause sentences (P <0.05). There was no significant difference between the two groups in comprehension of simple active, simple active with noun phrase and passive sentences (P> 0.05). They had a significant difference in auditory and visual reaction time (P <0.05). Also there was a significant difference between the two groups in syntactic priming and sentence completion tasks. However, in non-canonical condition of sentence completion, the difference between the two groups was not significant (P> 0.05).

**Conclusion:**

The results of the present study showed that the mean scores related to comprehension, production and working memory in people with mild Alzheimer’s were lower than healthy aged people, which indicate sentence processing problems at this level of the disease. People with Alzheimer have difficulty comprehending and producing complex syntactic structures and have poorer performance in tasks that required more memory demands. It seems that the processing problems of these people are due to both working memory and language problems, which are not separate from each other and both are involved in.

## Introduction

Alzheimer’s disease is the most common type of dementia, which is clinically identified with cognitive problems [1] memory, especially working memory [2], language skills, and inability to perform daily activities and behavioral problems [3]. Linguistic disorders as the main and predominant symptom in the early and preclinical stages of Alzheimer’s disease in recent years have been considered as one of the important diagnostic parameters in Alzheimer’s disease diagnosis protocols [4, 5]. The pattern of these linguistic disorders is heterogeneous and different in each patient but visible from the early and even preclinical stages [6]. Language disorders are progressive in Alzheimer’s and cover different language domains [7]. Among these language disorders confrontational naming, information retrieval, verbal paraphrasing especially in conversational contexts, abstract language comprehension difficulties, difficulty comprehending complex sentences, deficits in semantic and syntactic processing, simplification of the pattern of syntactic structures and sometimes difficulties in sentence processing are obvious [8]. In this article, our focus is on syntax processing. In the following, we will investigate the production and comprehension of syntax in AD patients, and since working memory plays an important role in these categories, the relationship between working memory and comprehension and production of sentences will be discussed.

Patients’ speech is fluent and some morphological-syntactic errors such as omission of functional words are visible which can indicate problems in sentence processing. Syntactic processing, in general, is the process at which different types of information are utilized in order to construct the grammatical structure of a sentence. Parsing is one of the important parts of syntactic processing at which a syntactic structure is assembled from a string of words [9]. However, semantic processing is basically concerned with retrieving word meanings. Semantic processing derives the organization of verbal information for both storage and retrieval [10]. Sentence processing in AD patients is influenced by numerous grammatical and meta-grammatical factors such as sentence structures, verb components, sentence components, characteristics of the verbs, and also the processing capacity of each person [11]. Comprehension and production in Alzheimer’s patients are examined from two aspects of lexical-semantic processing and syntactic processing. Although studies agree that Alzheimer’s patients have difficulty in comprehending and expressing sentences [12, 13], there is no consensus on the underlying problem of this disorder: Older studies have attributed speech and comprehension processing problems to damage in semantic representations [14, 15], while recent studies suggested that damages in comprehension and sentence production in Alzheimer’s patients are attributed to impaired performance in syntactic processing [11, 16]. Although the results of some studies indicate that syntactic processing is not a problem, at least in the early and mild stages of Alzheimer’s disease [17–19], some studies suggest that there are syntactic simplifications and short sentence length which indicate syntactic problems in these patients [20–29]. Patients with Alzheimer’s disease may have language problems in the early stages of the disease, but the prominent disorder is episodic memory and the progression of the disease leads to a reduction in other cognitive areas. Linguistic deficits are mainly due to the reduced lexical semantic abilities along with naming problems and semantic paraphrases, speech comprehension and verbal fluency problems [18, 30, 31]. As the disease progresses, language problems become more prevalent in Alzheimer’s patients, and their speech is limited to echolalia and verbal stereotypes [32].

Choi in 2019 compared normal elderly people with Alzheimer’s disease and found that this group of patients have impaired sentence comprehension and impairment in comprehension is strongly influenced by the syntactic complexity of sentences in a way that as sentence complexity increases, comprehension decreases sharply and slowing down speech in sentences with high syntactic complexity cannot improve perception in these patients [33].

Difficulty in producing sentences is another deficit in patients with Alzheimer’s disease. Some studies (e.g., 34) reported that the production of basic and simple sentences (routine) in the early stages of the disease is much worse than more advanced stages and people are weaker to produce simple sentences in the early stages [34].

Another known deficit in Alzheimer’s patients is a deficit in working memory which plays an important role in processing comprehension and expression. The results of studies on the relationship between working memory and sentence processing in Alzheimer’s patients are different and two different perspectives are observed. In the first view, it is believed that poor performance in the central execution mechanism of working memory has a positive correlation with sentence processing problems, especially sentences with prepositions, and in fact sentence processing problems are due to memory deficits, not language deficits [35–37]. Given the importance of working memory in comprehension problems, Kempler, Almor, Tyler, Andersen and MacDonald suggested that interventions use some techniques to increase working memory capacity instead of focusing on language structures, as working memory deficits reduce a person’s ability to maintain active representations of the information needed to process pronouns [38].

It has also been shown that the use of working memory interventions in Alzheimer’s patients leads to an increase the ability to produce language [39]. On the other hand, the results of some studies indicate that working memory deficit in Alzheimer’s patients occurs at the mild stage of the disease and also the syntax and comprehension of complex sentences are damaged in the moderate stages of the disease. Therefore, the main reason for comprehension problems is different according to the severity of the disease [40].

Can et al. in 2017 studied patients with Alzheimer’s disease in the primary and secondary stages and found that the length of sentences in these patients is shortened from the early stages and processes related to the sentence length are changed and this is affected by limitations in working memory [41]. In addition to the length of the sentences, Sajjadi et al. in 2012 showed that even in the early stages of the disease, Alzheimer’s patients tend to use less complex and simple syntactic structures [26].

But in the second view, the syntactic processing of sentences alone underlies the problem in Alzheimer’s patients and the problem of syntactic processing has linguistic roots. In principle, in this view, the positive relationship between memory function and language is not considered and language and memory are considered as two separate components. In this case, in these patients, parameters of the language may not be impaired while working memory is impaired [33, 42]. Studies have shown that Alzheimer’s patients have difficulty processing sentences, especially when verbs become more complex and have multiple prepositions [43]. It has also been shown that Alzheimer’s patients have a major syntactic problem that is not related to semantic or dysfunctional working memory problems, and that they only have difficulty comprehending when the demand for working memory resources increases, even if the semantic information in the sentence adequate comprehension [42].

Early detection is one of the most important challenges associated with Alzheimer’s disease. Currently, Alzheimer’s disease is diagnosed using postmortem brain images or specific diagnostic criteria and neurological examinations using tools such as MMSE or cognitive instruments such as Moca [27]. Looking for non-invasive, low-cost, and non-adversarial approaches to diagnosing and classifying Alzheimer’s patients, researchers are trying to use speech analysis and linguistic parameters such as lexical richness, lexical-syntactic diversity, word-to-speech ratio, and MMSE score to help in preclinical stages [44–46]. Linguistic markers such as telegraphic speech, prominently agraphia, repetitiveness of questions and statement, more writing errors, declines in structural complexity of the utterances, and semantic impairments can be helpful in predicting the onset of AD [47].

In recent years, the study of syntactic and semantic features of language have been used not only to diagnose Alzheimer’s in preclinical stages but also to determine the degree of dementia such as the Clinical Dementia Rating (CDR) test [48]. Researchers have also considered speech analysis to differentiate between people with Alzheimer’s and the elderly using machine learning-based approaches that examine the semantic, syntactic, and pragmatic features of language as an innovative context for Alzheimer’s research [49]. In the same line, Sung in 2020 found out that passive structures can be the best predictor to efficiently distinguish the mild cognitive impairment (MCI) group from the normal aging group. Since linguistic complexity plays a really important role in the detection of early emerging symptoms of linguistic-cognitive decline, what she stated has both theoretically and clinically importance for the field [50].

In summary, from a linguistic point of view and based on the results of studies, AD patients have difficulty processing complex syntactic sentences as well as non-canonical language structures. In Persian, non-canonical sentences are considered as marked sentences and need more processing load. In the present study, we also used canonical and non-canonical sentences. Considering that the novelty of the present study lies in the recognition that syntactic processing patterns and working memory and their relationship may be a diagnostic parameter in the early and preclinical stages of Alzheimer’s disease [51], this study examines two objectives. First, we aim to investigate sentence processing patterns in two levels of comprehension and production in mild AD patients compared to the healthy aged people. Any difference observed between the two groups may signal the possibility that comprehension and production tasks can be considered as helpful in diagnostic protocols of AD. Our second purpose is to shed more lights to understand the main cause of sentence processing problems based on working memory deficits. For this purpose, we will manipulate working memory load by using tasks which are designed in different memory-demanding levels. In this respect, one of the tasks demands more and the other demands fewer working memory resources. Regarding the clinical and theoretical importance of the matter, if part of the syntax processing that depends on memory changes, but processing without the involvement memory does not change for a person, this provides evidence that syntactic processing problems in AD patients may be due to working memory deficits. It is also important to note that working memory is compared in both groups.

## Experiments

The study includes 3 experiments; a sentence production task, a sentence comprehension task and a working memory experiment. Sentence comprehension experiment included 2 different tasks: a sentence-picture matching and a cross modal naming task. Sentence production experiment also included 2 different tasks: syntactic-priming and sentence completion tasks. Before the Experiments, 50 healthy aged people were tested for calculating the validity and reliability of the tasks. To measure the reliability of tests such as syntactic-priming, sentence completion and sentence-picture matching tasks which included questions with correct or incorrect answers, the Kuder-Richardson method was used. Cronbach’s alpha reliability test was also used for cross modal naming task which had quantitative answers. The reliability of the syntactic priming task was 0.8, and 0.79 for sentence-completion task. For sentence-picture matching task, the reliability was 0.82, and the reliability of the cross-modal naming task was 0.85. Then 25 healthy elderly people and 25 patients with mild Alzheimer’s were included in the study to perform the main test. Each experimental session lasted approximately 45 minutes. The experiment was conducted in a quiet room. They were given a set of 10 practice trials including samples of all conditions in order to familiarize themselves with the experimental tasks. Subjects were given a 5-minute break between tasks. All pictures were printed on A4 screen in white background. The laptop was used only for performing cross modal naming task.

## Method

### Participants

A total of 25 healthy elderly people (4 women, 21 men) with the age range of 61 to 77 and 25 patients with mild AD (9 women, 16 men) with the age range of 60 to 80 attended in this study. All participants were evaluated by two geriatricians using the Diagnostic and Statistical Manual Disorders-Fourth Edition (DSM-IV) Criteria for the Diagnosis of Alzheimer’s Disease and National Institute of Neurological and Communicative Disorders and Stroke and the Alzheimer’s Disease and Related Disorders Association (NINCDS-ADRDA) Criteria. Also the Persian Clinical Dementia Rating (P-CDR) scale was conducted for all of the participants [52]. According to CDR scores, although the score of healthy old people was 0, the score for patients with mild AD ranged from 0.5 to 1. Inclusion criteria for study were: being Persian native speakers, having normal hearing and vision and having no history of psychiatric disorders or other cognitive problems. In order to assess the working memory capacity, the forward and backward digit span tasks from Wechsler Adult Intelligence Scale-IV (WAIS) were used [53]. The tasks included sequences of numbers starting from span 2 to span 8. Participants were required to repeat the numbers in forward or backward chains after hearing them. Both directions included 14 trials. If the participant could not recall both trials for each span, the testing process was stopped. The total score for each task was 14 and the whole score for working memory task was 28. Demographic information of participants is presented in Table 1. In this study, as determined by t and p-values, individuals in the two groups were matched in terms of age (t (50) = -1, p = 0.07), and education (t (50) = 1, p = 0.09). Due to some limitation in accessing participants during the Coronavirus pandemic, there was no matching in the case of gender.

**Table 1:** Demographic information of the participant.

	Age Mean ± SD	Education Mean ± SD	DF Mean ± SD	DB Mean ± SD
AD (n = 25)	70.68±6.638	11±2	7.44±1.917	3.76±1.562
NC (n = 25)	67.12±5.357	12±2	9.64±1.868	5.76±1.877

*Note*. Demographic information for patients with Alzheimer’s disease (AD) and normal controls (NC), digit forward (DF), digit backward (DB), age at the time of testing and years of education.

### Ethics approval

Ethics committee approved the whole procedure of Isfahan University of Medical Sciences (*Ethics code*: *IR*.*MUI*.*RESEARCH*.*REC*.*1398*.*304*). Participants completed the formal written ethical consent form of Isfahan University of Medical Sciences.

## Experiment 1: Sentence production task

We used two different tasks for the Sentence production section: Sentence-completion task and syntactic priming task. The sentence-completion task requires less memory demands than the syntactic-priming task. Since the important components of the sentence are written below the image and the examiner provides the beginning of the sentence, the sentence-completion task is assumed to be less dependent on working memory. A syntactic priming task is a memory-demanding task which is based on syntactic-priming paradigm. The participants should memorize the syntactic structure they hear and then produce the syntactic structure similar to it. Since participants have to keep the syntactic features of the sentence in memory, it seems that this task is more memory-demanding than the sentence-completion task [51]. We considered task demands in two ways: (a) memory requests, by promoting two controlled sentence-production tasks: a sentence-completion task and a syntactic-priming task, in addition (b) linguistic computational loads by differing the canonicity of a word order. Focusing on syntactic features by decreasing semantic processing with a limited vocabulary is the role of these sentence-production tasks [51].

### Procedure

#### Syntactic-priming task

**Fig 1:**
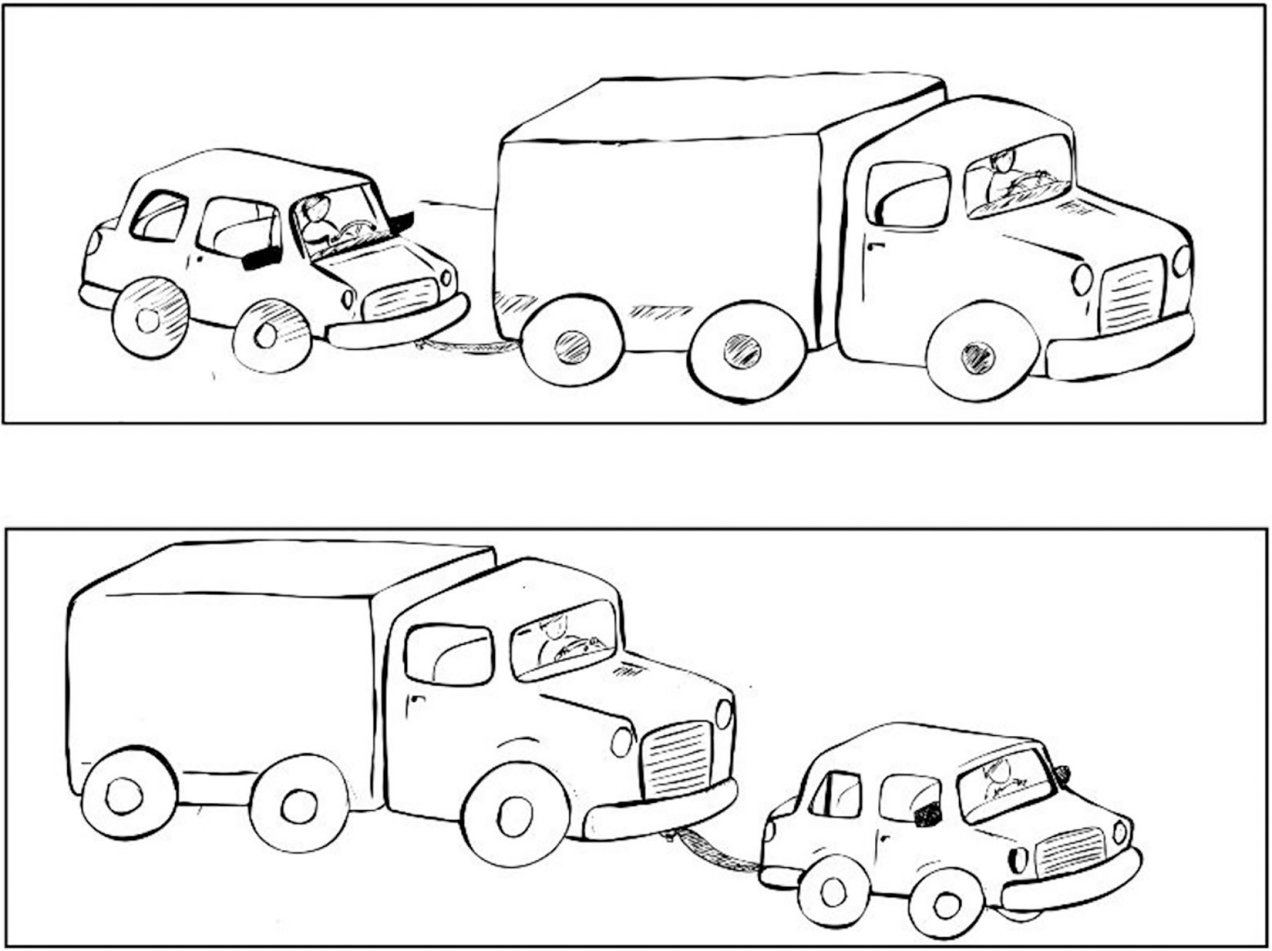
An example of a syntactic priming task.

Each trial included two pictures: one picture was used as the prime picture and the other was the target picture. The examiner produced a passive sentence including an agent, a patient, and an action about the prime picture. The participants kept the sentence in their memories and then produced a sentence with the same structure in order to describe the target picture. All verbs used in sentences were transitive verbs [51]. For instance, examiners produced; “the truck is pulled by the car”, then asked participants to produce a sentence for the target picture by saying” the car is pulled by the truck” (See Fig 1). The experiment consisted of two different conditions: In condition 1, the passive-canonical condition, the noun phrase was placed at the head of a sentence followed by a by-phrase (tavasote). (e.g., “mashin tavasote kamiun keshide shod” meaning “The car by the truck was pulled.”). In condition 2, the passive-non-canonical condition, the noun phrase was placed at the head followed by the verb phrase including a by-phrase (tavasote). (e.g., “mashin keshide shod tavasote kamiun” meaning “The car was pulled by the truck.”).

The responses were scored correct when participants formed a passive sentence with correct grammatical markers for every thematic role. If participants did not use a prime target syntactic structure, responses were scored as an error.

#### Sentence-completion task

**Fig 2:**
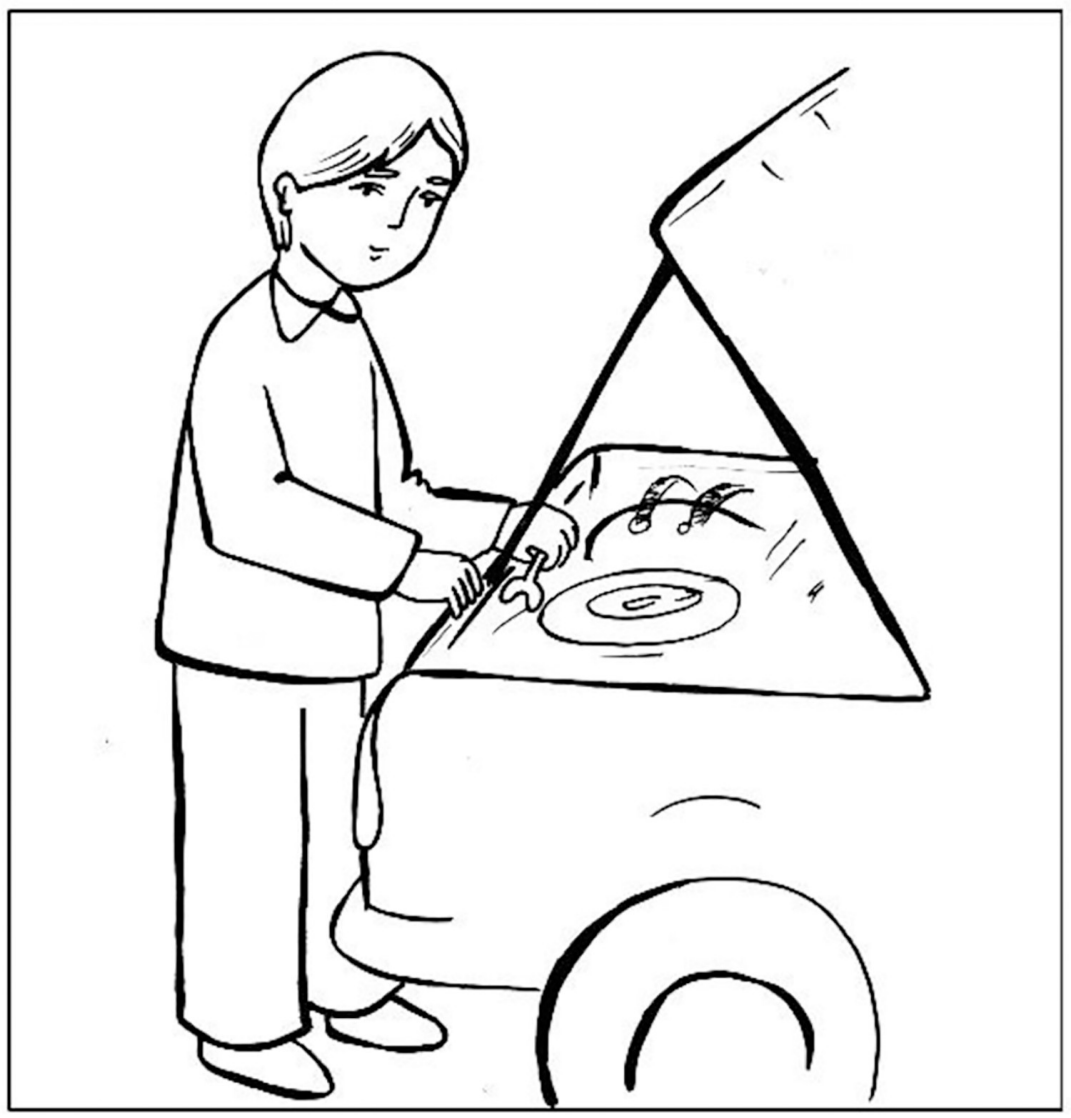
An example of a sentence-completion task.

This task included pictures in which a verb and a noun were written below them (See Fig 2). The participants were asked to complete the first noun phrase using either a nominative or a dative (by-phrase) grammatical marker that was presented by the examiner. Similar to the previous task, there were two conditions on the canonicity [51]. In condition 1, a canonical condition, examiners first initiated the sentence with a noun phrase and a "by" phrase assigned to the patient and asked participants to complete the rest of the sentence. Participant had to use a passive form, to correctly explain the picture: (e.g., “gol tavasote dokhtar bu shod” meaning “the flower by the girl was smelled”). In condition 2, a non-canonical condition, examiners initiated a noun phrase and asked participants to complete the statement with by-phrase. (e.g., “Mashin tamir shod tavasote pesar” meaning “the car was repaired by the boy”).

### Results

The sentence production ability in syntactic-priming task was compared between the AD and normal individuals using independent t-test. The mean score passive-canonical syntactic-priming in AD patients with M = 14.16 were significantly different from normal individuals with M = 14.92 (t (48) = 2.06>1.96, p<0.05). Also in passive-non-canonical syntactic-priming task, the mean score in AD patients (M = 9) was significantly (t (48) = 4.56>1.96, p<0.001) less than the mean score in normal individuals (M = 14.28). In the sentence-completion task, AD patients (M = 14.72) had no significant difference (t (48) = 1.37<1.96, p>0.1) with normal individuals (M = 15) in passive-canonical conditions. But in passive-non-canonical conditions, the mean score in AD patients (M = 13.32) was significantly (t (48) = 2.72>1.96, p<0.05) less than the mean score in normal individuals (M = 14.88).

## Experiment 2: Sentence comprehension tasks

For assessing sentence comprehension, we used sentence-picture matching protocol task which is a more memory-demanding task than the cross-modal naming test.

### Procedure

#### Sentence-picture matching

This task included 40 sentences, using four grammatical constructions: simple active, active with a conjoined noun phrase (NP), full passive, and relative clause (subject-subject and object-subject relatives). Sentences varied in terms of number of words (ranged from 5 to 9 words), the number of components (2 vs. 3) and syntactic complexity and included nouns and transitive verbs. The number of sentence components in each grammatical construction increased. The main reason for this procedure was to make the sentences more complex in terms of visual representation and semantic density of the picture. Two pictures depicted above all stimuli one of which served the role of a distracting factor which contained the same participants who played the same action in reversed roles. To put it differently, all the grammatical structures used in this experiment were reversible. For instance, the target picture depicted the action “the girl is pushed by the boy” which was followed by a distracting picture depicting the action “the boy is pushed by the girl”.

After listening to each sentence presented by the researcher, the subjects had to point to one of the two pictures which printed on A4 screen in front of them that best showed the intended meaning of the sentence. Different hypotheses about the source of the deficits in participants’ comprehension could be tested using these sentences structures. For example, if comprehension problems were due to the syntactic complexity, the participants should perform poorly on structures like relative clauses and active with a conjoined NP in comparison to simple active sentences [38].

#### Cross-modal naming

Twenty grammatical and ungrammatical sentences were used for the purpose of this cross-modal naming test, from which 10 stimuli were played auditory followed by a visual presented target and the other 10 stimuli were represented visually followed by a visual presented target. All of the sentences began with a complete context sentence followed by an incomplete sentence which needs an appropriate verb to be grammatically and semantically correct. The stimuli were played one after another which was soon followed by a target verb appearing on the screen of laptop in front of them. This target verb was either grammatically correct or ungrammatical. The laptop used in this study was Asus model K555L and also tsco2399 speaker was used for playing the auditory stimuli. Participants were sitting 50 cm apart from the monitor. All texts appeared in font 72 in a fixed location in the center of the screen. The volume of the loudspeakers was load enough for the listeners so that they could clearly listen the stimuli. The subjects were asked to listen to the auditory stimuli or read the visual stimuli simultaneously and then read the target verb attentively [38]. Afterwards, the subjects were asked to judge whether the appeared target verb could grammatically complete the sentence by answering using the words “correct” and “incorrect”.

Data collection and item presentation were done by DMDX softwareV.5.1.4.4. For each sentence reaction time (RT) was measured from the onset of item presentation to the beginning of participant response. After that, all of the output data of DMDX were imported CHECKVOCAL software to calculate exact RTs. In this part corrects and/or no responses were utilized for determining RT.

### Results

The sentence comprehension ability in sentence-picture matching task was compared between the AD and normal individuals using independent t-test. In the relative clause construction, AD patients with Mean = 8.16 performed significantly worse than the normal individuals with Mean = 9.76 (t (48) = 4.38>1.96, p<0.001) but there was no significant difference between AD and normal individuals in the other three grammatical constructions (simple active, active with a conjoined noun phrase (NP), and full passive, all with p>0.05). Furthermore, in cross modal naming task, the mean visual reaction time for AD patient’s M = 2451.256 was significantly higher than the mean time for normal individual with M = 1712.733 (t (48) = 5.02>1.96, p<0.001). Also mean auditory reaction time in AD patients (M = 2194.084) was significantly (t (48) = 5.21>1.96, p<0.001) different from normal individual (M = 1611.199).

### Statistical analysis

**Fig 3:**
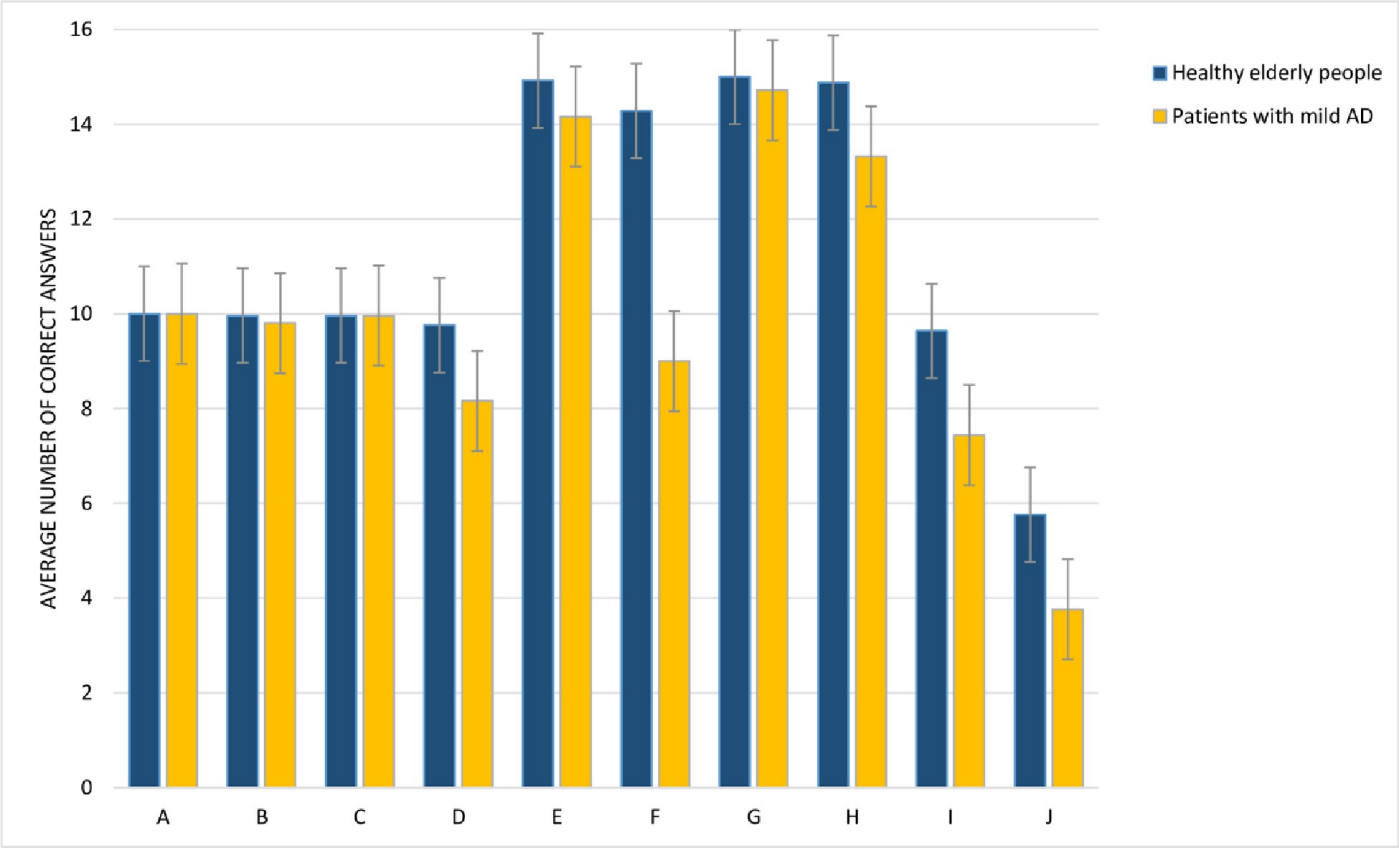
Note. A = comprehension of simple active; B = comprehension of simple active with noun phrase (NP); C = comprehension of full passive; D = comprehension of relative clause; E = production of canonical syntactic-priming; F = production of non-canonical syntactic-priming; G = production of canonical sentence-completion; H = production of non-canonical sentence-completion; I = forward digit span; J = backward digit span.

SPSS software (22) was used for the analyses of the scores. The T-test at the 95% confidence level and the Pearson correlation test were used for statistical analyses and calculating the correlation between sentence comprehension, sentence production, and working memory in the Alzheimer and aged people group, respectively (See Fig 3).

## Discussion

The present study compared the comprehension and production of sentences in the mild stage of the disease. The choice of more or less dependent tasks on working memory assisted us to determine to some extent that 1) Is the source of comprehension and sentence processing problems in Alzheimer’s patients linguistic or cognitive? And 2) Can syntax processing be used to help diagnose Alzheimer’s disease early?

Supported by many other studies, the findings of the present study showed that AD patients have problems in working-memory, especially in backward digit span, which is identified from the early stages of the disease [54, 55]. Considering the fact that in backward digit span both storing and processing of information occur, this task needs more cognitive or processing resources. The phonological loop capacity is involved in both forward digit span and backward digit span, while the latter also involves the central executive component of working memory [56]. Previous research revealed that hypo perfusion, regional frontal loop degeneration, and also the breakdown of effective connectivity between neuronal system leads to working-memory difficulties [57].

As mentioned earlier, four sentence structures, namely simple-active, simple-active with noun phrase (NP), full passive, and relative clause, were used in assessing the sentence-picture matching task. AD patients showed only problems in comprehension of relative clause. Our findings were not in line with Kempler’s study. In his study mild AD patients had difficulties in comprehending relative clauses in addition to the other three structures [38]. To put it differently, AD patients had problems in comprehension of more complex syntactic structure. Relative clauses are the most important and complex descriptive sub-sentence, which is more complex than other constructions both in terms of syntax and number of words and sentence length. Perhaps this syntactic complexity and the increase in sentence length, and thus the increase in memory load, have made it difficult for Alzheimer’s people to understand relative clauses. AD patients did not show difficulties in comprehending simple active and simple active with NP structures which may be due to the high frequency of these structures in Persian and their simple syntactic structures. Our findings in comprehending syntactically-complex structures including two clauses (such as relative clauses), were in line with what Rochon, Just, Carpenter, and Choi have earlier reported [33, 35, 58]. The findings in comprehending passive structures, however, was quite in contrast to what Sung et.al have recently reported. Sung used sentence-picture paradigm with semantically reversible sentence and showed that patients with cognitive problems such as mild cognitive impairments (MCI) have difficulty comprehending passive structures, and these passive sentences were considered as the most important predictors in distinguishing the MCI group from the normal aging group [50]. Quite the contrary, in this study there was no difference between the normal aging group and AD patients, which have poorer cognitive skills than MCI group. This difference could also be due to differences between Korean and Persian.

In addition to different performance in sentence-picture matching, AD patients performed differently in cross-modal naming task, which is based on grammatical judgments. Research showed that cross-modal naming task is less memory-demanding compared to sentence-picture matching [38]. Since the reaction time is important in the analysis of this task, we have analyzed RTs. Participants were required to do grammatical judgment within 5000 milliseconds. If a person’s grammatical judgment lasted more than 5,000 milliseconds, it was considered as “no response”, however, the no response was also taken into account in calculating the reaction time [59]. In this study, the mean reaction time in normal aged group was 1644 milliseconds, and 2315 milliseconds in AD patients. In other words, the more “no response” answers in AD patients led to a significant difference in their reaction time compared to the other group. This is consistent with the results reported in Kempler in 1998. In his study, the mean reaction time of normal aged group was 2300 milliseconds, however, the mean RT was more than 4000 in AD patients. This reduction in the speed of sentence processing may lead to comprehension problems, as stated in [13]. Therefore, the increase in the reaction time may be related to the reduction in the speed of sentence processing. The speed of language processing is a cognitive skill [60] and since AD patients have cognitive problems, the weak performance in cross-modal naming task seems to be justifiable.

As observed in the present study, AD patients exhibited significantly different in comparison to the normal aging group in syntactic priming task, in both canonical and non-canonical conditions and also in non-canonical sentence completion task, but not in the canonical sentence completion task. Syntactic priming task is more memory demanding than sentence completion task and previous studies in language processing reported that syntactic priming is based on the non-declarative memory. The neuroimaging also shows that left inferior frontal gyrus (IFG), bilateral supplementary motor area (SMA), and left middle temporal area are all involved in processing the language and the sequencing syllable structure, and in the retrieval of lexical syntactic information from memory [61]. Regarding the results from the syntactic priming task in this study, the AD patients were supposed to substitute the subject and object in addition to keeping the syntactic and semantic information embedded in the sentence. The patients’ failure in substitution and manipulation of the syntactic structure of the sentence may cause them difficulties in performing syntactic priming task which may be due to the lack of residual effect and memory impairment in them [62].

Non-canonical sentence completion tasks require more linguistic and cognitive processing resources in comparison to the canonical sentence completion tasks. Although the length of the sentences was the same in both canonical and non-canonical conditions, the non-canonical condition is syntactically more complex, and therefore, it was cognitively more demanding in production [51]. The sentence completion task is less memory demanding, and it was expected that the AD patients perform quit well in it. However, due to the more complex syntactic structures in the non-canonical sentence completion, they performed weakly in this task.

The results of this study were in line with the results of the study of Bates et al. They examined the production of complex syntax, and found that people with Alzheimer’s disease had difficulty producing passive sentences and performed weakly in the descriptions of the two events and instead, produced simpler forms that could easily reflect memory problems in these patients [63]. On the other side of the desk, Can and Kuruoglu [34] stated that AD patients’ syntactic ability in sentence production is often intact and deficits are just observed in complex sentences, but it is ultimately the stage of the disease which determines the severity of language problems, and syntactic problems show themselves in the moderate and severe stages. Nonetheless in the present study mild AD patients also showed problems in syntactic processing. It is noteworthy that the aging group did not perform that well in syntactic priming and sentence completion tasks which may be as a result of the decrease in the processing resources.

## Conclusion

One of the main question was whether the syntactic processing patterns can be used as an early diagnosis of Alzheimer’s disease. From a linguistic point of view, as expected, AD patients’ performance in processing complex linguistic constructions as well as non-canonical sentence was weak. We found that relative clauses and sentences with non-canonical condition can be helpful in early diagnosis of the disease. The effect of syntactic priming was also weak in AD patient’s production which may be due to the lack of residual effect and memory impairment in them. However, more research is required to be conducted. Regarding the second challenge in the field that whether comprehension and production processing problems have cognitive (memory) or linguistic (syntactic) sources, the present study showed that both of them are involved in creating comprehension and production problems and that they are not separated from each other. Thus, this study does not support the hypothesis of working memory impairments. Because the working memory impairment hypothesis suggests that sentence processing problems in AD patients are only due to the memory problems and have no linguistic sources. Of course, there is still a need for more studies in this field.

### Limitation

- Unavailability of Alzheimer’s patients and aged people due to the coronavirus epidemic.
- In this study sex was not controlled and we suggest that gender be controlled in future studies.

## Supporting information

S1 Data (SAV)

## References

[1] Mace NL, Rabins PV. The 36-hour day: A family guide to caring for people who have Alzheimer disease, other Dementias, and memory loss: JHU Press; 2017.

[2] Dronkers NF, Wilkins DP, Van Valin RD Jr, Redfern BB, Jaeger JJJC. Lesion analysis of the brain areas involved in language comprehension. Cognition. 2004;92(1–2):145–77. 15037129 10.1016/j.cognition.2003.11.002

[3] Bahro M, Silber E, Sunderland TJJotAGS. How Do Patients with Alzheimer’s Disease Cope with Their Illness?‐A Clinical Experience Report. 1995;43(1):41–6. doi: 10.1111/j.1532-5415.1995.tb06240.x 7806738

[4] Brandt J, Bakker A, Maroof DA. Auditory confrontation naming in Alzheimer’s disease. The Clinical Neuropsychologist. 2010;24(8):1326–38. doi: 10.1080/13854046.2010.518977 20981630 PMC2992092

[5] Lin C-Y, Chen T-B, Lin K-N, Yeh Y-C, Chen W-T, Wang K-S, et al. Confrontation naming errors in Alzheimer’s disease. Dement Geriatr Cogn Disord. 2014;37(1–2):86–94. doi: 10.1159/000354359 24107364

[6] Slegers A, Filiou R-P, Montembeault M, Brambati SM. Connected speech features from picture description in Alzheimer’s disease: A systematic review. Alzheimer’s Dis. 2018;65(2):519–42. doi: 10.3233/JAD-170881 30103314

[7] Friedmann N, Gvion AJB, Language. Sentence comprehension and working memory limitation in aphasia: A dissociation between semantic-syntactic and phonological reactivation. Brain Lang. 2003;86(1):23–39. doi: 10.1016/s0093-934x(02)00530-8 12821413

[8] Marková J, Horváthová Ľ, Králová M, Cséfalvay Z. Sentence comprehension in Slovak‐speaking patients with Alzheimer’s disease. Int J Lang Commun Disord. 2017;52(4):456–68. doi: 10.1111/1460-6984.12284 28000389

[9] Field J. Psycholinguistics: The key concepts. 1st ed. Psychology Press; 2004.

[10] de Leede-Smith S, Roodenrys S, Horsley L, Matrini S, Mison E, Barkus E. Role for Positive Schizotypy and Hallucination Proneness in Semantic Processing. Front Psychol. 2020;11:2272. doi: 10.3389/fpsyg.2020.542002 32982899 PMC7492677

[11] Small JA, Kemper S, Lyons K. Sentence repetition and processing resources in Alzheimer’s disease. Brain Lang. 2000;75(2):232–58. doi: 10.1006/brln.2000.2355 11049667

[12] Friederici AD, Meyer M, von Cramon DYJB, language. Auditory language comprehension: an event-related fMRI study on the processing of syntactic and lexical information. Brain Lang. 2000;74(2):289–300. doi: 10.1006/brln.2000.2313 10950920

[13] Grossman M, Rhee J. Cognitive resources during sentence processing in Alzheimer’s disease. Neuropsychologia. 2001;39(13):1419–31. doi: 10.1016/s0028-3932(01)00059-8 11585610

[14] Martin A, Fedio P. Word production and comprehension in Alzheimer’s disease: The breakdown of semantic knowledge. Brain Lang. 1983;19(1):124–41. doi: 10.1016/0093-934x(83)90059-7 6860932

[15] Altmann LJ. Constrained sentence production in probable Alzheimer disease. Appl. Psycholinguist. 2004;25(2):145. doi: 10.1017/S0142716404001080

[16] Bickel C, Pantel J, Eysenbach K, Schröder J. Syntactic comprehension deficits in Alzheimer’s disease. Brain Lang. 2000;71(3):432–48. doi: 10.1006/brln.1999.2277 10716871

[17] Kavé G, Levy Y. Morphology in picture descriptions provided by persons with Alzheimer’s disease. J Speech Lang Hear Res. 2003 2003;46(2):341–52. doi: 10.1044/1092-4388(2003/027 14700376

[18] Forbes-McKay KE, Venneri A. Detecting subtle spontaneous language decline in early Alzheimer’s disease with a picture description task. Neurol Sci. 2005;26(4):243–54. doi: 10.1007/s10072-005-0467-9 16193251

[19] Tang-Wai DF, Graham NL. Assessment of Language Function in Dementia. Geriatrics and Aging. 2008;11(2):103–10.

[20] Altmann LJ, Kempler D, Andersen ES. Speech errors in Alzheimer’s disease: reevaluating morphosyntactic preservation. J Speech Lang Hear Res. 2001; 44(5):1069–82. doi: 10.1044/1092-4388(2001/085) 11708528

[21] Ripich DN, Carpenter BD, Ziol EW. Conversational cohesion patterns in men and women with Alzheimer’s disease: a longitudinal study. Int J Lang Commun Disord. 2000;35(1):49–64. doi: 10.1080/136828200247241 10824224

[22] Kemper S, LaBarge E, Ferraro FR, Cheung H, Cheung H, Storandt M. On the preservation of syntax in Alzheimer’s disease: Evidence from written sentences. Arch Neurol. 1993;50(1):81–6. doi: 10.1001/archneur.1993.00540010075021 8418805

[23] Boschi V, Catricala E, Consonni M, Chesi C, Moro A, Cappa SF. Connected speech in neurodegenerative language disorders: a review. Front Psycho. 2017;8:269. doi: 10.3389/fpsyg.2017.00269 eCollection 2017. 28321196 PMC5337522

[24] Ash S, Moore P, Vesely L, Grossman M. The decline of narrative discourse in Alzheimer’s disease. Brain Lang. 2007;103:181–2. doi: 10.1016/j.bandl.2007.07.105

[25] De Lira JO, Ortiz KZ, Campanha AC, Bertolucci PHF, Minett TSC. Microlinguistic aspects of the oral narrative in patients with Alzheimer’s disease. Int. Psychogeriatr. 2011;23(3):404. doi: 10.1017/S1041610210001092 20699046

[26] Sajjadi SA, Patterson K, Tomek M, Nestor PJ. Abnormalities of connected speech in semantic dementia vs Alzheimer’s disease. Aphasiology. 2012;26(6):847–66. doi: 10.1080/02687038.2012.654933

[27] Orimaye SO, Wong JS-M, Golden KJ, editors. Learning predictive linguistic features for Alzheimer’s disease and related dementias using verbal utterances. Proceedings of the workshop on computational linguistics and clinical psychology: From linguistic signal to clinical reality; 2014.

[28] Ash S, Grossman M. Why study connected speech production? In R. Willems (Ed.), Cognitive Neuroscience of Natural Language Use (pp. 29–58). Cambridge: Cambridge University Press. (2015). doi: 10.1017/CBO9781107323667.003

[29] Yancheva M, Fraser KC, Rudzicz F, editors. Using linguistic features longitudinally to predict clinical scores for Alzheimer’s disease and related dementias. Proceedings of SLPAT 2015: 6th Workshop on Speech and Language Processing for Assistive Technologies; 2015.

[30] Taler V, Phillips NA. Language performance in Alzheimer’s disease and mild cognitive impairment: a comparative review. J Clin Exp Neuropsychol. 2008;30(5):501–56. doi: 10.1080/13803390701550128 18569251

[31] Catricalà E, Della Rosa PA, Plebani V, Vigliocco G, Cappa SF. Abstract and concrete categories? Evidences from neurodegenerative diseases. Neuropsychologia. 2014;64:271–81. doi: 10.1016/j.neuropsychologia.2014.09.041 25281886

[32] Ferris SH, Farlow M. Language impairment in Alzheimer’s disease and benefits of acetylcholinesterase inhibitors. Clin Interv Aging. 2013;8:1007. doi: 10.2147/CIA.S39959 23946647 PMC3739421

[33] Choi H, Choi H. Ability of sentence comprehension according to syntactic complexity and speech rate in patients with Dementia of Alzheimer’s Type. CSD. doi: 10.12963/csd.19602

[34] Can E, Kuruoglu G. A Comparison of Sentence Production of Turkish Pat? ents w? th Early and Late-Onset Alzheimer’s Disease. Psycho-Educational Research Reviews. 2018:74–85–74–85.

[35] Rochon E, Waters GS, Caplan D. The relationship between measures of working memory and sentence comprehension in patients with Alzheimer’s disease. J Speech Lang Hear Res. 2000;43(2):395–413. doi: 10.1044/jslhr.4302.395 10757692

[36] Alatorre-Cruz GC, Silva-Pereyra J, Fernández T, Rodríguez-Camacho MA, Castro-Chavira SA, Sanchez-Lopez J. Effects of age and working memory load on syntactic processing: an event-related potential study. Front Hum Neurosci. 2018;12:185. doi: 10.3389/fnhum.2018.00185 29780314 PMC5945836

[37] Norris LJ. Neural Synchrony During Naturalistic Language Perception in Listeners with Aphasia (Doctoral dissertation, University of South Carolina).2021.

[38] Kempler D, Almor A, Tyler LK, Andersen ES, MacDonald MC. Sentence comprehension deficits in Alzheimer’s disease: a comparison of off-line vs. on-line sentence processing. Brain Lang. 1998;64(3):297–316. doi: 10.1006/brln.1998.1980 9743544

[39] Lee MS, Kim BS. Effects of working memory intervention on language production by individuals with dementia. Neuropsychol Rehabil. 2020:1–25. doi: 10.1080/09602011.2020.1789479 32677586

[40] Liu X, Wang W, Wang H, Sun Y. Sentence comprehension in patients with dementia of the Alzheimer’s type. PeerJ. 2019;7:e8181. doi: 10.7717/peerj.8181 31824775 PMC6896939

[41] Can E, Kuruoglu G, Yener G, Özsoy AS. Sentence length of Turkish patients with early and late-onset Alzheimer’s disease. Humanit. soc. sci. rev. 2017;6(02):69–78.

[42] Grober E, Bang S. Sentence comprehension in Alzheimer’s disease. Dev Neuropsychol. 1995;11(1):95–107. doi: 10.1080/87565649509540606

[43] Waters GS, Caplan D, Rochon E. Processing capacity and sentence comprehension in patients with Alzheimer’s disease. Cogn. Neuropsychol. 1995;12(1):1–30. doi: 10.1080/02643299508251990

[44] López-de-Ipiña K, Alonso J-B, Travieso CM, Solé-Casals J, Egiraun H, Faundez-Zanuy M, et al. On the selection of non-invasive methods based on speech analysis oriented to automatic Alzheimer disease diagnosis. Sensors. 2013;13(5):6730–45. doi: 10.3390/s130506730 23698268 PMC3690078

[45] Ammar RB, Ayed YB. Language-related features for early detection of Alzheimer Disease. Procedia Comput. Sci. 2020;176:763–70. doi: 10.12963/csd.19602

[46] Szatloczki G, Hoffmann I, Vincze V, Kalman J, Pakaski M. Speaking in Alzheimer’s disease, is that an early sign? Importance of changes in language abilities in Alzheimer’s disease. Frontiers in aging neuroscience. 2015;7:195. doi: 10.3389/fnagi.2015.00195 26539107 PMC4611852

[47] Eyigoz E, Mathur S, Santamaria M, Cecchi G, Naylor M. Linguistic markers predict onset of Alzheimer’s disease. EClinicalMedicine. 2020;28:100583. doi: 10.1016/j.eclinm.2020.100583 33294808 PMC7700896

[48] Lai Y-h Pai H-h. To be semantically-impaired or to be syntactically-impaired: Linguistic patterns in Chinese-speaking persons with or without dementia. J. Neurolinguistics. 2009;22(5):465–75. doi: 10.1016/j.jneuroling.2009.03.004

[49] Ammar RB, Ayed YB, editors. Speech processing for early Alzheimer disease diagnosis: machine learning based approach. 2018 IEEE/ACS 15th International Conference on Computer Systems and Applications (AICCSA); 2018: IEEE.

[50] Sung JE, Choi S, Eom B, Yoo JK, Jeong JH. Syntactic complexity as a linguistic marker to differentiate mild cognitive impairment from normal aging. J Speech Lang Hear Res. 2020;63(5):1416–29. doi: 10.1044/2020_JSLHR-19-00335 32402217

[51] Sung JEJPo. Age-related changes in sentence production abilities and their relation to working-memory capacity: evidence from a verb-final language.2015;10(4):e0119424. doi: 10.1371/journal.pone.0119424 25856161 PMC4391780

[52] Sharifi K. Diagnostic accuracy comparison between the Persian versions of clinical dementia rating (P-CDR) and cognitive state test (P-COST) in the elderly dementia screening. Nurs Midwifery J. 2016; 14(6): 551–561.

[53] Wechsler D. Wechsler adult intelligence scale. Archives of Clinical Neuropsychology. 1955.

[54] MacDonald MC, Almor A, Henderson VW, Kempler D, Andersen ES. Assessing working memory and language comprehension in Alzheimer’s disease. Brain Lang. 2001;78(1):17–42. doi: 10.1006/brln.2000.2436 11412013

[55] Perpetuini D, Chiarelli AM, Filippini C, Cardone D, Croce P, Rotunno L, et al. Working memory decline in alzheimer’s disease is detected by complexity analysis of multimodal EEG-FNIRS. Entropy. 2020;22(12):1380. doi: 10.3390/e22121380 33279924 PMC7762102

[56] Ruchinskas R. Wechsler adult intelligence scale-digit span performance in subjective cognitive complaints, amnestic mild cognitive impairment, and probable dementia of the Alzheimer type. Clin Neuropsychol. 2019;33(8):1436–44. doi: 10.1080/13854046.2019.1585574 30931811

[57] Huntley J, Howard R. Working memory in early Alzheimer’s disease: a neuropsychological review. International Journal of Geriatric Psychiatry: Int J Geriatr Psychiatry. 2010;25(2):121–32. doi: 10.1002/gps.2314 19672843

[58] Just MA, Carpenter PA. A capacity theory of comprehension: individual differences in working memory. Psychol Rev. 1992;99(1):122. doi: 10.1037/0033-295x.99.1.122 1546114

[59] Moayedfar S, Purmohammad M, Shafa N, Shafa N, Ghasisin L. Analysis of naming processing stages in patients with mild Alzheimer. Appl Neuropsychol Adult. 2021;28(1):107–116. doi: 10.1080/23279095.2019.1599894 31030561

[60] Ebaid D, Crewther SG, MacCalman K, Brown A, Crewther DP. Cognitive processing speed across the lifespan: beyond the influence of motor speed. Front Aging Neurosci. 2017;9:62. doi: 10.3389/fnagi.2017.00062 eCollection 2017. 28381999 PMC5360696

[61] Heyselaar E, Segaert K, Walvoort SJ, Kessels RP, Hagoort P. The role of nondeclarative memory in the skill for language: Evidence from syntactic priming in patients with amnesia. Neuropsychologia. 2017;101:97–105. doi: 10.1016/j.neuropsychologia.2017.04.033 28465069

[62] Kootstra GJ. Code-switching in monologue and dialogue: Activation and alignment in bilingual language production: [Sl: sn]; 2012.

[63] Bates E, Harris C, Marchman V, Wulfeck B, Kritchevsky M. Production of complex syntax in normal ageing and Alzheimer’s disease. Lang Cogn Process. 1995;10(5):487–539. doi: 10.1080/01690969508407113

